# Risk and protection of different rare protein-coding variants of complement component C4A in age-related macular degeneration

**DOI:** 10.3389/fgene.2023.1274743

**Published:** 2024-01-29

**Authors:** Johanna M. Seddon, Dikha De, William Casazza, Shun-Yun Cheng, Claudio Punzo, Mark Daly, Danlei Zhou, Samantha L. Coss, John P. Atkinson, Chack-Yung Yu

**Affiliations:** ^1^ Department of Ophthalmology and Visual Sciences, University of Massachusetts Chan Medical School, Worcester, MA, United States; ^2^ Broad Institute of MIT and Harvard, Cambridge, MA, United States; ^3^ Abigail Wexner Research Institute, Nationwide Children’s Hospital, Columbus, OH, United States; ^4^ Department of Internal Medicine, Division of Rheumatology, Washington University School of Medicine, Saint Louis, MO, United States

**Keywords:** age-related macular degeneration, complement system, C4A, C4B, complement proteins, genetic associations, targeted sequencing

## Abstract

**Introduction:** Age-related macular degeneration (AMD) is the leading cause of central vision loss in the elderly. One-third of the genetic contribution to this disease remains unexplained.

**Methods:** We analyzed targeted sequencing data from two independent cohorts (4,245 cases, 1,668 controls) which included genomic regions of known AMD loci in 49 genes.

**Results:** At a false discovery rate of <0.01, we identified 11 low-frequency AMD variants (minor allele frequency <0.05). Two of those variants were present in the complement *C4A* gene, including the replacement of the residues that contribute to the Rodgers-1/Chido-1 blood group antigens: [VDLL1207-1210ADLR (V1207A)] with discovery odds ratio (OR) = 1.7 (*p* = 3.2 × 10^−5^) which was replicated in the UK Biobank dataset (3,294 cases, 200,086 controls, OR = 1.52, *p* = 0.037). A novel variant associated with reduced risk for AMD in our discovery cohort was P1120T, one of the four C4A-isotypic residues. Gene-based tests yielded aggregate effects of nonsynonymous variants in 10 genes including *C4A*, which were associated with increased risk of AMD. In human eye tissues, immunostaining demonstrated C4A protein accumulation in and around endothelial cells of retinal and choroidal vasculature, and total C4 in soft drusen.

**Conclusion:** Our results indicate that C4A protein in the complement activation pathways may play a role in the pathogenesis of AMD.

## 1 Introduction

Age-related macular degeneration (AMD) is a complex neurodegenerative disease. It is the leading cause of central vision loss in elderly individuals. The disease is characterized by reduced function of retinal pigment epithelium (RPE) and photoreceptor cell loss in the macula. Advanced AMD is classified as wet when accompanied by neovascularization involving the choroidal and/or retinal vasculature or the dry, atrophic form called geographic atrophy. These late stages of the disease are typically preceded by clinically asymptomatic earlier stages. AMD affects more than 20 million people in the United States, of which 1.5 million people are estimated to have advanced stages (Rein et al., 2022). At present, the pathobiology of the disease, especially the atrophic stage, is not well understood, and treatment for this advanced dry form is in its early stages.

Genetic and environmental factors contribute to the development of AMD (Seddon, 2017). Identifying genetic variants associated with AMD can help uncover disease mechanisms, aid in diagnosis, and provide insights into potential therapies. Genome-wide association studies (GWAS) of AMD cases and controls have identified common susceptibility variants in different loci and have uncovered multiple cellular pathways that are involved in AMD pathology. The AMD associated loci are within the genes *CFH, ARMS2, HTRA1, C2, CFB, C3, CFI, C9* and others (Klein et al., 2005; Maller et al., 2006; Maller et al., 2007; Fagerness et al., 2009; Neale et al., 2010; Raychaudhuri et al., 2011; Fritsche et al., 2013; Seddon et al., 2013; Zhan et al., 2013; Yu et al., 2014; Fritsche et al., 2016; Yu et al., 2016) in the immune, inflammatory, lipid, angiogenesis and DNA and cell repair pathways. However, translating these loci into biological insights remains a challenge as the functional consequences of disease-associated variants are typically subtle and hard to decipher.

With advances in next-generation sequencing technology and the accumulation of AMD samples, genetic analyses have been extended to search for rare variants. Compared to common variants that tag an associated genomic region, rare coding variants often have more obvious functional consequences, provide specific clues about the underlying molecular mechanism, and can thus more readily accelerate the translation from biological understanding to therapeutics. Identifying multiple disease-associated coding common and rare variants in the same genes provides strong evidence that disrupting gene function leads to disease development. Most studies that implicated specific rare variants in AMD either relied on exome-wide assessments or on targeted analyses of a few genes in a relatively modest numbers of individuals. Larger sample size would enhance identification of rare variants.

The first confirmed rare variant associated with AMD was *CFH* R1210C (rs121913059), a highly penetrant variant, with a frequency of 1.4% in AMD compared with less than 0.1% in control populations (Raychaudhuri et al., 2011). *CFH* R1210C was also associated with earlier age of onset of AMD, more rapid progression to advanced stages, and a typical fundus phenotypic appearance including high drusen burden in the macula and extramacular locations in both eyes (Seddon et al., 2014; Ferrara and Seddon, 2015; Seddon and Rosner, 2019; Seddon et al., 2020).

We also performed targeted sequencing of the exons in 681 genes that were either within reported AMD loci or in related pathways (Seddon et al., 2013). In this study (the Age-Related Maculopathy Targeted Sequencing or ARTS), we found additional new coding variants in loci that were associated with advanced AMD: rare variants that increased risk of AMD in *C3* (K155Q), *C9* (P167S) and a burden of *CFI* rare coding variants (Seddon et al., 2013). Another group performed a smaller targeted sequencing study of 10 known AMD risk loci consisting of 57 genes using data from Michigan, Mayo, AREDS, and Pennsylvania (MMAP) cohorts (Zhan et al., 2013). They reported the same *C3* rare variant, K155Q, and the previously discovered *CFH* variant, R1210C, but did not find the *CFI* rare variants or evaluate *C9.*

To identify additional novel rare variants, we combined the above two datasets to increase sample size and performed association analyses for overlapping targeted genomic regions, which included 10 AMD risk loci in 49 genes. We discovered intriguing antigenic variants that likely switched the blood group association from Rodgers-1 to Chido-1 in C4A (Yu et al., 1986; Yu et al., 1988) in AMD patients. We also performed immunostaining using a Rodgers-1 specific monoclonal antibody (mAb RgD1) to investigate the locations of C4A protein in normal human donor retinal tissue.

## 2 Materials and methods

### 2.1 Study overview

**FIGURE 1:**
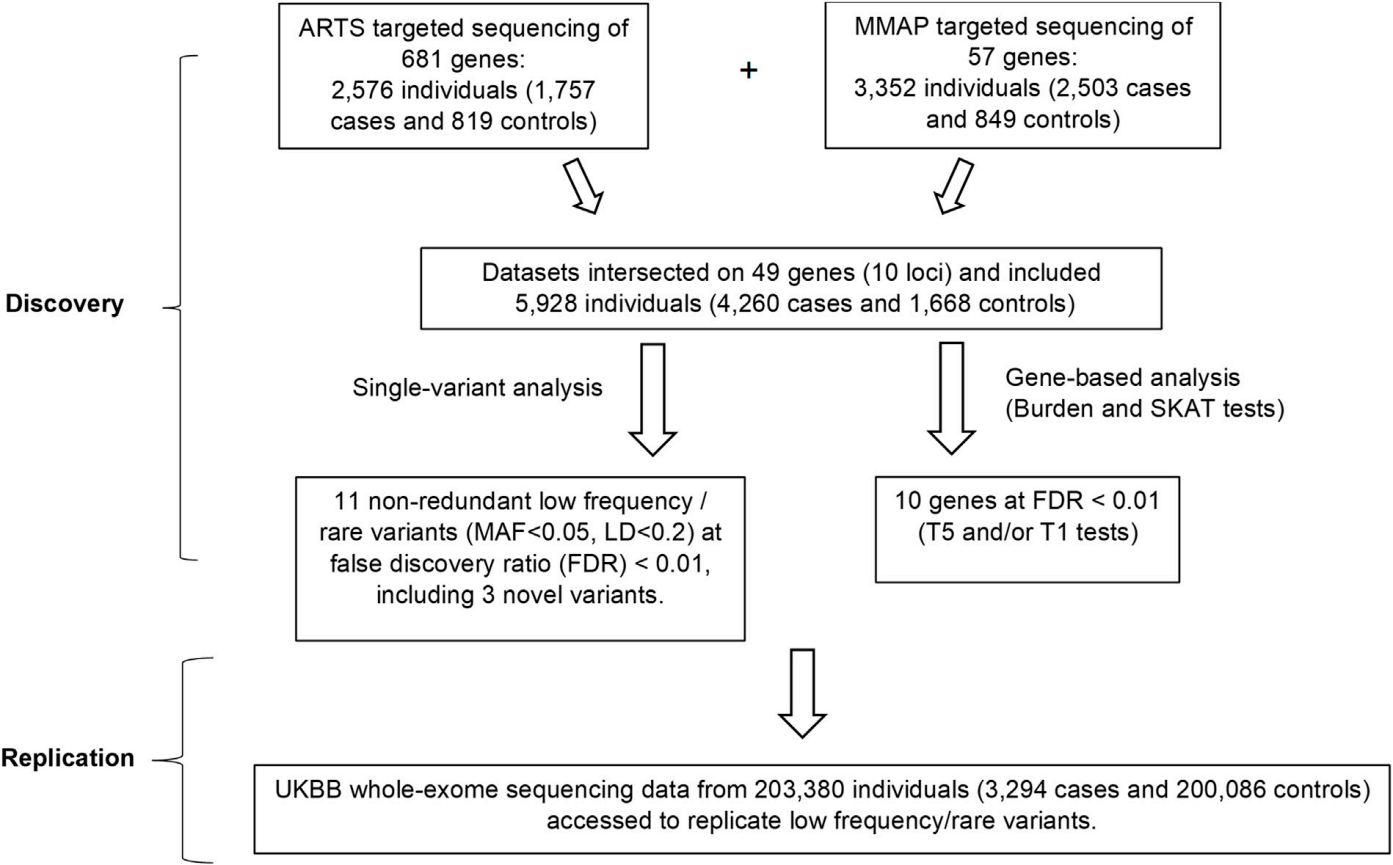
Overall study design. In the discovery phase, single-variant and gene-based analyses were performed by combining the data from the two cohorts. Associations of 3 rare or low-frequency variants (MAF<0.05) and 10 genes with AMD were identified at FDR<0.01. In the replication phase, these variants and genes were checked for their associations with AMD in the UKBB whole-exome sequencing data. ARTS = Age-Related Maculopathy Targeting Sequencing; MMAP = Michigan, Mayo, AREDS, Pennsylvania; MAF = Minor Allele Frequency; UKBB = UK Bio Bank.

The study design is shown in Figure 1. We combined data from two cohorts as the discovery set and focused on the overlapping targeted genomic regions in the two studies including AMD risk loci and candidate loci (in or near *CFH, ARMS2, C3, C2-CFB, CFI, CETP, LIPC and TIMP3-SYN3, LPL* and *ABCA1*) (Seddon et al., 2013; Zhan et al., 2013). Association analyses were performed for low-frequency and rare variants (at MAF < 0.05 in the 1,000 genomes reference panel) for which the minor allele occurred in at least 10 individuals. The significant novel results identified in the discovery set were then assessed in the UK Biobank whole exome sequencing (UKBB WES) dataset.

### 2.2 ARTS study

The Age-Related Maculopathy targeted sequencing study (ARTS) included 2,576 participants, 1,757 cases and 819 controls from the Seddon Longitudinal Cohort Study (Seddon et al., 2013; Ferrara and Seddon, 2015; Seddon, 2017; Seddon and Rosner, 2019; Seddon et al., 2023). Board-certified ophthalmologists evaluated cases and control subjects. All cases had intermediate or advanced AMD according to the Clinical Age-Related Maculopathy Grading System (CARMS): stage 3 (intermediate AMD), stage 4 (central or non-central geographic atrophy, GA) and stage 5 (choroidal neovascularization, NV) (Seddon et al., 2006). Controls were also examined and had no signs of intermediate or advanced macular degeneration in either eye and did not have bilateral early AMD. Most of the controls (>80%) were age >60 years old. All participants were of European ancestry. Genomic regions of 681 genes within reported AMD loci and related pathways were sequenced using a custom Agilent SureSelectXT kit to capture the genomic sequences of coding exons, splice junctions and 5′ UTR and 3′ UTR regions. A total target length of 5.28 Mb including 1.76 Mb of coding exons were sequenced with paired-end reads using an Illumina HiSeq 2000 sequencing platform. Samples included in the analysis had over 10× coverage at >90% of targeted regions and over 20× coverage at >80% of targeted regions.

### 2.3 MMAP study

This study included 3,337 participants (2,488 cases and 849 controls) from ophthalmology clinics at the University of Michigan (UMich), University of Pennsylvania (UPenn) and the Age-Related Eye Disease Study (AREDS) (Zhan et al., 2013). AMD cases were defined as having GA, NV, or large drusen in at least one eye. Controls were examined and had varying criteria including no more than 5 hard drusen and over age 50 or small drusen and pigment changes in one eye only and over age 60. Participants were of primarily European ancestry as computed with GRAF using ancestry informative SNPs (Jin et al., 2019). Genomic regions of 57 genes within 10 candidate loci of AMD risk were sequenced using the Illumina Genome Analyzer IIx and HiSeq 2000 platforms. Sequencing covered 80% of the targeted space at a depth of >20×, with an average of 123,221,974 bases per individual (127.5× average coverage). Within targeted regions, 98.49% of the protein-coding exons had coverage of >10×. The bam files of targeted sequencing data were downloaded from NIH dbGAP (https://www.ncbi.nlm.nih.gov/gap/) with accession number phs000684.

### 2.4 Read mapping and quality control

Sequence reads were aligned to the human reference genome (NCBI Build 37, GRCh37) with Burrows-Wheeler Aligner (BWA, v0.7.17). Genotypes in the target regions were called using GATK (v3.5) with the workflow and parameters recommended in best practice variant detection with GATK v3. We applied GATK duplicate removal, indel realignment and base quality score recalibration and performed multi-sample SNP and indel discovery and genotyping across all samples simultaneously using variant quality score recalibration (VQSR). Other than high-quality variants assigned “PASS” by VQSR, we annotated variants with snpEff and Annovar (Cingolani et al., 2012).

We excluded SNPs failing the Hardy-Weinberg test in controls (*p* < 1 × 10^−6^) and alleles that had high missing genotype data (>1%), likely owing to systematic low coverage or difficulty mapping reads across many samples. Samples with high missing genotype data (>1%) for common alleles with >1% frequency in each dataset were excluded. We also removed low-quality variants with average depth of <0.5 or >500; and variants with evidence of strand bias or cycle bias (PHRED score<50); and variant sites within 5 bp of a 1000 Genomes Project indel.

### 2.5 UKBB study

The UK Biobank (UKBB) project is a large-scale prospective cohort study of half a million participants across the United Kingdom, aged between 40 and 69 at the time of recruitment (2006–2010) (Szustakowski et al., 2021). UKBB WES data were available for 203,380 subjects. We identified 3,294 AMD cases using one of the following criteria: (1) ICD-9 or ICD-10 diagnosis codes (3625 and H353); (2) responded “Macular degeneration” in “eye problems/disorders”; (3) responded “Macular degeneration” in self-reported non-cancer illness. The remaining subjects were treated as controls.

### 2.6 Single variant association analysis

We identified 2,974 single nucleotide variants that had a minor allele frequency (MAF) <0.05 in 1,000 Genomes (or GnomAD if unavailable in 1,000 Genomes) and which had a minor allele present in at least 10 samples in the combined ARTS and MMAP cohorts. Of these variants, 418 were non-synonymous, 193 were synonymous, 103 were splice region variants, and the remainder were intronic. We used logistic regression, implemented in the *glm* function in R, to model the association between the number of minor alleles per subject and AMD status (0 or 1), adjusting for 15 genotype principal components calculated over variants with an MAF ≥ 0.05. At the discovery stage, we used a Benjamini-Hochberg false-discovery rate (FDR) of less than 0.01 to call out associated variants with suggestive significance. As a sensitivity analysis, we performed this same association analysis separately in the ARTS and MMAP datasets and meta-analyzed the two sets of associations using a fixed-effect meta-analysis implemented in the Meta R package (Schwarzer et al., 2015). In comparing the results of our pooled analysis to this fixed-effect meta-analysis, we demonstrate whether heterogeneity in effects for each study influenced significant associations called in our discovery analysis. In the presence of heterogeneity, we expect the results of a pooled analysis and a fixed-effect meta-analysis to disagree. However, we observed an agreement in Odds Ratios (ORs) between our pooled analysis and fixed effect meta-analysis for each rare variant associated with AMD status at an FDR < 0.01, with a minimum heterogeneity *p* > 0.08 using Cochran’s Q (Supplementary Table S1). Since both sex and age were available in ARTS, we also tested whether adjusting for age and sex in our logistic regression model affected the association of variants detected in our pooled analysis. Similarly, we found negligible differences in their association with AMD status.

### 2.7 Identify independent SNPs

Pairwise LD among the SNPs was calculated to detect any potential independent signals. SNPs with the lowest *p* values in association with AMD in each LD block at *r* ^*2*^ < 0.2 were selected for further examination. We curated a list of known AMD risk SNPs including the SNPs collected by GWAS Catalog at *P* < 5 × 10^−8^, or by ClinVar annotated by “macular degeneration” category. Conditional analysis was performed by conditioning on the known AMD risk SNPs in the same loci, and the SNPs with conditional *p* < 0.05 were considered as novel independent AMD risk SNPs. For SNPs in *C4*, conditional analysis also included the variants associated with *C4A* copy number.

### 2.8 Molecular biologic studies of complement *C4* including *C4A* copy number variants and phenotypes

Using DNA samples from 8 selected AMD patients with *C4* variants from the Seddon Longitudinal Cohort Study, gene copy number (GCN) variations of total *C4* (*C4T*), *C4A*, *C4B*, long genes (*C4L*) and short genes (*C4S*) of complement *C4* were assayed by TaqMan-based real time PCR using five independent amplicons as described previously (Wu et al., 2007). Validation of GCN data were confirmed when *C4T* = *C4A*+*C4B* = *C4L*+*C4S*. Complement C4A and C4B protein phenotypes were determined using EDTA-plasma, which were subjected to neuraminidase and carboxyl peptidase B digestion and then resolved using high voltage agarose gel electrophoresis. C4A and C4B protein allotypes were detected and stained after immunofixation (Chung et al., 2005). To confirm sequence variations at the C4d region, 2.5 kb genomic DNA fragments corresponding to exon 22 and exon 30 were amplified from selected patients with AMD and then purified and cloned into TA-cloning vector. Plasmid clones with C4d fragments together with appropriate sequencing primers were sent to Eurofin for Sanger sequencing. Polymorphic variants were analyzed by comparing genomic DNA sequences of *C4A* and *C4B* genes as described (Yu, 1991; Zhou et al., 2021).

### 2.9 Burden and SKAT analyses

The gene-level analysis was used to evaluate aggregate effects from rare and low frequency variants at MAF <5% (T5 test) or MAF<1% (T1 test) in a gene for AMD using the standard burden test and Sequence kernel association testing (SKAT), implemented in SKAT R package (Wu et al., 2011). Nonsynonymous variants for each gene were grouped for tests. Covariates included 15 principal components. Burden tests assume that all variants in a gene either increase or decrease disease risk. SKAT allows for variants with opposite directions of effect to reside in the same gene.

### 2.10 Functional annotation of variants

We used Combined Annotation Dependent Depletion (CADD) (Rentzsch et al., 2019) and PolyPhen-2 (Adzhubei et al., 2010) to predict functions of identified variants. A scaled CADD score of 20 means that a variant is amongst the top 1% of deleterious variants in the human genomes. PolyPhen-2 score ranged from 0 to 1. Variants with scores closer to 1 in PolyPhen-2 are more confidently predicted to be deleterious.

### 2.11 Immunostaining of C4A in human retinal tissues

Donor tissues were obtained from the Eye Donation Project under the protocol of JMS that was approved by the Institutional Review Board (IRB) of the University of Massachusetts Chan Medical School. The superior quadrants of each eye were fixed in 4% paraformaldehyde, embedded in optimal cutting media and then processed for cryo-sectioning. Dissection, fixation and embedding of human eye tissue were performed as done for mouse tissue, and was processed for immunohistochemistry as described previously (Cheng et al., 2020). Retina was sectioned at 20 µm thickness, permeabilized in PBS+0.3% Triton X-100 at RT. The primary Ab used was a mouse anti-*C4A* (1:300, anti-RgD1, kindly provided by Dr. Joanne Moulds). For the immunohistochemistry staining with a secondary donkey anti-mouse IgG HRP (1:500, Jackson ImmunoResearch; Cat no: 715-036-151) was employed, with the ImmPACT VIP kit (Vector Laboratories, Cat no: SK-4605) for detection. Primary and secondary Abs were diluted in PBS with 0.3% Triton X-100% and 5% bovine serum albumin (BSA, Cell Signaling Technology). Incubation of primary Ab was performed over night at 4°C, while incubation of secondary Ab was performed at RT for 2 h. All images were visualized with a Leica DM6 Thunder microscope utilizing or employing a 12-bit color camera.

## 3 Results

The study design is outlined in Figure 1. We focused on the low frequency, rare variants with a minor allele frequency (MAF) <0.05 and identified 11 independent variants (linkage disequilibrium [LD] <0.2) in association with AMD at a false discovery rate (FDR) of <0.01 (Table 1). Among the 11 variants, 3 were novel (i.e., they were not related to any reported AMD risk variant with LD threshold *r* ^*2*^ < 0.2). These findings were then evaluated for replication in the UK Biobank (UKBB) cohort (Szustakowski et al., 2021) [including 3,294 cases and 200,086 controls employing whole-exome sequencing (WES)].

**TABLE 1:** Rare or low-frequency genetic variants associated with AMD.

Marker	Chr	Position	Gene	Function/Protein change	ALT/REF	MAF in 1000 genomes	Discovery cohort ^*^		Direction of effect on AMD	Replication cohort ^ǂ^	
							OR (CI)	*p* value		OR (CI)	*p* value
*NOVEL*											
rs966136477	1	196621093	*CFH*	5′UTR	T/A	N/A	0.55 (0.4, 0.73)	7.34E-05	−		
rs201206908	6	31963859	*C4A*	P1120T	A/C	0.0048	0.26 (0.14, 0.66)	3.90E-04	−, −	0.90 (0.24, 2.34)	0.55
rs28357075	6	31964321	*C4A*	V1207A	C/T	0.0081	1.7 (1.33, 2.2)	3.16E-05	+, +	1.52 (0.96, 2.30)	0.037
*KNOWN* (*LD r* ^*2*^ *>0.2 with previously reported AMD risk SNPs*)											
rs121913059	1	196716375	*CFH*	R1210C	T/C	N/A	8.85 (2.16, 36.69)	2.64E-04	+, +	6.6 (1.3, 22)	0.013
rs41310132	1	196928188	*CFHR2*	Y264C	G/A	0.012	0.44 (0.31, 0.63)	2.14E-06	−, −	1.0 (0.0, 1.2)	0.51
rs17201144	6	31730568	*MSH5*	UTR	G/A	0.043	0.7 (0.59, 0.84)	9.12E-05	−		
rs9332739	6	31903804	*C2*	E318D	C/G	0.03	0.53 (0.42, 0.64)	7.79E-10	−, −	1.1 (0.98, 1.2)	0.09
rs4151670	6	31915532	*CFB*	Y726Y	T/C	0.013	0.47 (0.35, 0.61)	3.71E-08	−, −	0.84 (0.69, 0.98)	0.032
rs61746206	6	32065113	*TNXB*	A173T	T/C	0.042	0.47 (0.33, 0.67)	1.40E-05	−, −	0.85 (0.69, 1.0)	0.047
rs79021034	10	124216821	*ARMS2*	3′UTR	T/G	N/A	5.05 (3.31, 8.11)	2.95E-15	+		
rs147859257	19	6718146	*C3*	K155Q	G/T	0.0004	5.25 (2.45, 11.43)	2.35E-06	+, +	1.5 (1.1, 2.0)	0.008

* Discovery cohort refers to pooling of two cohorts: ARTS (Age-Related Maculopathy targeted sequencing study) and MMAP (Michigan, Mayo, AREDS, Pennsylvania).

ǂ Replication cohort refers to data derived from the UK Biobank. Blank indicates data not available in the datasets.; N/A: not available (indicating MAF of those variants was not shown in 1,000 genomes reference panel); ALT: alternate allele; Chr: Chromosome; MAF: minor allele frequency; REF: reference allele; OR: Odds Ratio; CI: Confidence Interval; LD: Linkage Disequilibrium.

### 3.1 Identification of novel rare or low frequency variants in association with AMD

Single-variant association tests were performed on 2,974 variants occurring in at least 10 samples, including 2,564 variants at MAF < 0.05 (MAF based on 1,000 genomes reference panels), in which the genomic control lambda was 0.92. The 10 loci sequenced in both ARTS and MMAP were known AMD risk loci. We focused on low-frequency, common and rare variants at MAF<0.05 in our analyses.

At a FDR of <0.01 (corresponding to P < 4 × 10^−4^), we identified 77 rare or low-frequency variants in association with AMD including 32 variants residing in exons, UTRs and splice junctions. Among these 32 variants, there were 11 non-redundant variants (pruned by LD *r* ^*2*^ < 0.2), as shown in Table 1, including 8 known variants that were previously reported or in LD (*r* ^*2*^ > 0.2) with reported AMD associated variants. The other 3 variants were novel variants (not associated with any known AMD risk variants or variants in LD with these risk variants at *r* ^*2*^ > 0.2, see Methods). Of the 3 novel variants, rs28357075 (p.V1207A) in the C4A protein was identified at a higher frequency in AMD cases than in controls (i.e., risk variant, odds ratio [OR] = 1.7, *p* = 3.16 × 10^−5^). The other 2 were protective variants for AMD including rs966136477 in 5′UTR of *CFH* (OR = 0.55, *p* = 7.34 × 10^−5^) and rs201206908 (P1120T) in C4A (OR = 0.26, *p* = 3.9 × 10^−4^). The frequency of this C4A mutation (P1120T) in AMD cases was 0.24% (10/4,260) and in controls was 0.90% (15/1,668).

### 3.2 Replication of novel variants associated with AMD in UKBB WES cohort

**FIGURE 2:**
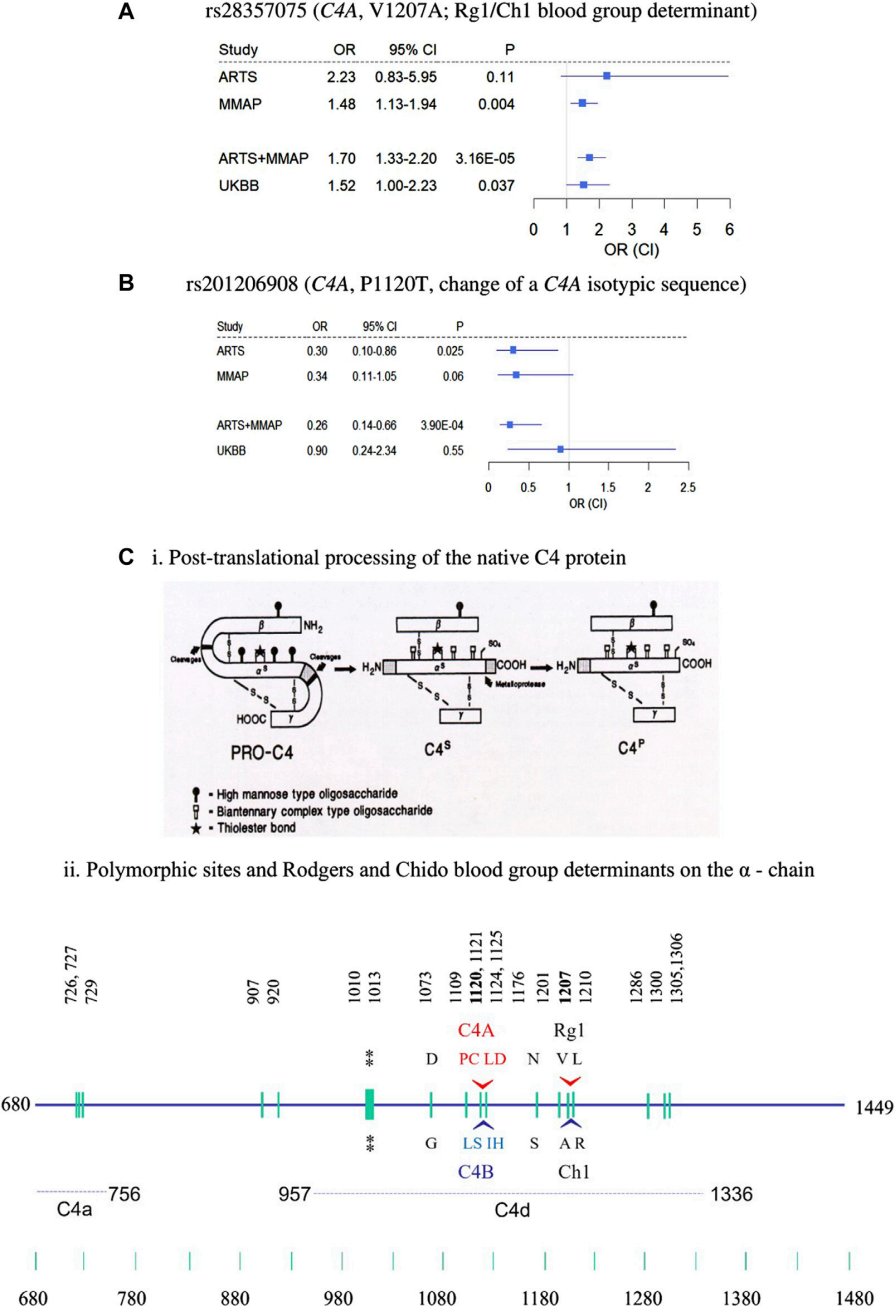
Nonsynonymous rare variants in C4A. **(A–B)** forest plots of rs28357075 **(A)** and rs201206908 **(B)** showing odds ratio estimates and confidence intervals. **(C)** Structural diversities of human C4 proteins. (i) post-translational processing of C4 protein from biosynthesis (PRO-C4) to secretion (C4^s^) to plasma (C4^p^) forms in the circulation. Sites for the high mannose type and biantennary types of glycosylations are shown. The thioester bond is shown by a star. (ii) Polymorphic sites on the α-chain highlighting locations and sequences specific for the C4A and C4B isotypes, and the antigenic determinants of the Rodgers (Rg) and Chido (Ch) blood groups, whose major determinants are Rg1 and Ch1, respectively. The location of the thioester bond is shown by double asterisks. The precursor C4 protein is synthesized as a single chain molecule with 1744 residues and processed to a three-chained structure (beta-alpha-gamma) linked by disulfides. Numbering of amino acids is based on the initiation codon for the C4 precursor protein as number 1 (Chan et al., 1984; Yu et al., 1988; Yu et al., 2002).

Of the 3 novel variants described above, none were previously reported in AMD, including in the full summary statistics of AMD GWAS by the International AMD Genomics Consortium (Fritsche et al., 2016). We checked for these variants in the UKBB WES data including 3,294 cases and 200,086 controls (Table 1). The nonsynonymous variant in *C4A*, rs28357075 (V1207A, MAF = 0.0081, discovery OR = 1.7, *p* = 3.16 × 10^−5^) was replicated in UKBB (replication OR = 1.52, *p* = 0.037). Figures 2A, B show forest plots of the two *C4* variants in ARTS, MMAP and UKBB. The change of Rodgers-1 related residue to Chido-1 (V1207A) in C4A increased the risk of AMD. The other *C4A* variant, (P1120T, rs201206908), was protective in the combined discovery cohorts (OR = 0.26, *p* = 3.9 × 10^−4^), but was not significantly associated with AMD in the UKBB cohort (OR = 0.90, *p* = 0.55).

### 3.3 *C4A* rs28357075 is statistically independent of *C4A* copy number variants

One study suggested that higher *C4A* gene copy number was protective against AMD and was driven by rs429608 at this same locus (Grassmann et al., 2016). The SNP rs429608 is an intronic variant in *SKIV2L* (Dangel et al., 1995; Yang et al., 1998), which has been reported to be associated with AMD (Fritsche et al., 2016). *C4A* rs28357075 (V1207A) is not in LD with rs429608 in the UKBB population at *r* ^*2*^ = 0.003. The targeted sequencing data of ARTS and MMAP were mainly focused on coding variants and did not assess rs429608.

To determine whether the association of rs28357075 (V1207A) with AMD was independent of the *C4A* copy number variation, we performed conditional analysis including a proxy SNP of rs429608 (i.e., rs641153 missense variants in *CFB*, D′ = 0.96 and *r* ^*2*^ = 0.67) in the logistic regression model. The association of rs28357075 with AMD remained statistically significant, conditioning on rs641153 (*p* = 9.28 × 10^−6^). We further tested *C4A* copy number variants and known AMD risk variants in this locus by conditional analysis (see Methods). Our results show that the association of rs28357075 with AMD is statistically independent of any known AMD variant and *C4A* copy number variant (Supplementary Tables S2, 3). On a separate note, while the A1207 for Ch1 always goes with R1210 (i.e., L1210R; Supplementary Table S2), we observed another variant with H1210 (L1210H). This is a novel rare variation, an example of a tri-allelic variation at the same specific location. Such a phenomenon may implicate a potential functional relevance at the location of Rg1/Ch1 epitope to drive structural diversity.

### 3.4 Gene-based analysis

Gene-based tests were conducted to assess aggregation of low-frequency or rare nonsynonymous or splicing variants with MAF<0.05 (T5 test) or <0.01 (T1 test) in a gene effect on AMD. At FDR<0.01, we identified 10 genes in association with AMD by T5 tests, and 3 genes (*CFH*, *C3*, and *C4A*) remained significant by T1 tests (Table 2). Among the 10 genes, 7 were found in other gene-based analyses (*C3*, C2, *CFB*, *CFH*, *MSH5*, *CFHR2,* and *SKIVL2*) (Fritsche et al., 2016; Yu et al., 2016). The gene-based result of *CFH* (*P*_SKAT_ = 0.003) was replicated in the UKBB.

**TABLE 2:** Genes associated with AMD in gene-based analysis.

Gene	Chromosome	Number of variants	*p*-value (burden)	*p*-value (SKAT)	T1 or T5
CFHR2*	1	6	0.11	4.34E-06	T5
CFH*	1	22	2.41E-04	7.65E-10	T1/T5
MSH5*	6	9	1.28E-04	1.47E-05	T5
C2*	6	11	9.91E-06	5.47E-08	T5
CFB*	6	12	7.85E-06	3.42E-12	T5
SKIV2L*	6	8	9.38E-03	1.12E-07	T5
*DXO*	6	8	0.53	2.90E-04	T5
*C4A*	6	5	0.01	7.05E-06	T1/T5
*TNXB*	6	70	3.10E-08	2.68E-08	T5
C3*	19	17	0.02	4.92E-06	T1/T5

Analyses are based on nonsynonymous variants only; T1 or T5: test had SKAT *p*-value < 0.05 based on allele frequency<0.01 (T1) or 0.05 (T5). For tests passing this threshold at both T1/T5 we report the *p*-value for T1.

*Indicates genes previously reported to be associated with AMD.

MSH5, C2, CFB, SKIV2L, DXO, C4A, TNXB are present in the class III region of the HLA (Carroll et al., 1984; Zhou et al., 2019).

CFHR2: Complement Factor H Related 2; CFH: Complement Factor H; MSH5: MutS Homolog 5; C2: Complement Component 2; CFB: Complement Factor B; SKIV2L: SKI2 subunit of Superkiller Complex; DXO: decapping exoribonuclease; C4A: Complement Component 4A (Rodgers Blood Group); TNXB: Tenascin XB; C3: Complement Component 3.

### 3.5 Inferred function of *C4A* variants

The above results highlighted the association of *C4* with AMD. Gene-based tests suggested the aggregated effects of 5 nonsynonymous variants in *C4A* contribute to AMD risk (*P*_*SKAT*_ = 7.05 × 10^−6^). The C4 protein (1744 amino acids) comprises three chains, the β-chain (20-675), α-chain (680-1,446), and the γ-chain (1,454-1,744). Among the 5 variants, 3 were risk variants for AMD (OR>1.0) including R916Q (rs148571233), V1207A (rs28357075) and L1210R (rs28357076), and 2 were protective variants (OR<1) including A1062V (rs768510893), and P1120T (rs201206908) (Supplementary Table S2). All 5 nonsynonymous variants were in the α-chain and independent of each other at LD *r* ^*2*^ < 0.01 (Figure 2C). PolyPhen-2 predicted the P1120T substitution as “probably damaging” with a score of 0.995, and a CADD score = 19.18, and the other four were predicted as “benign”. As a reminder, PolyPhen-2 classifies substitutions solely based on benign vs. damaging effects, and CADD scoring is based on conservation, which is only a proxy for deleteriousness. Thus, this annotation may reflect that this substitution affects C4A protein function, which may emphasize the importance of P1120T for further evaluation as a protective variant.

**FIGURE 3:**
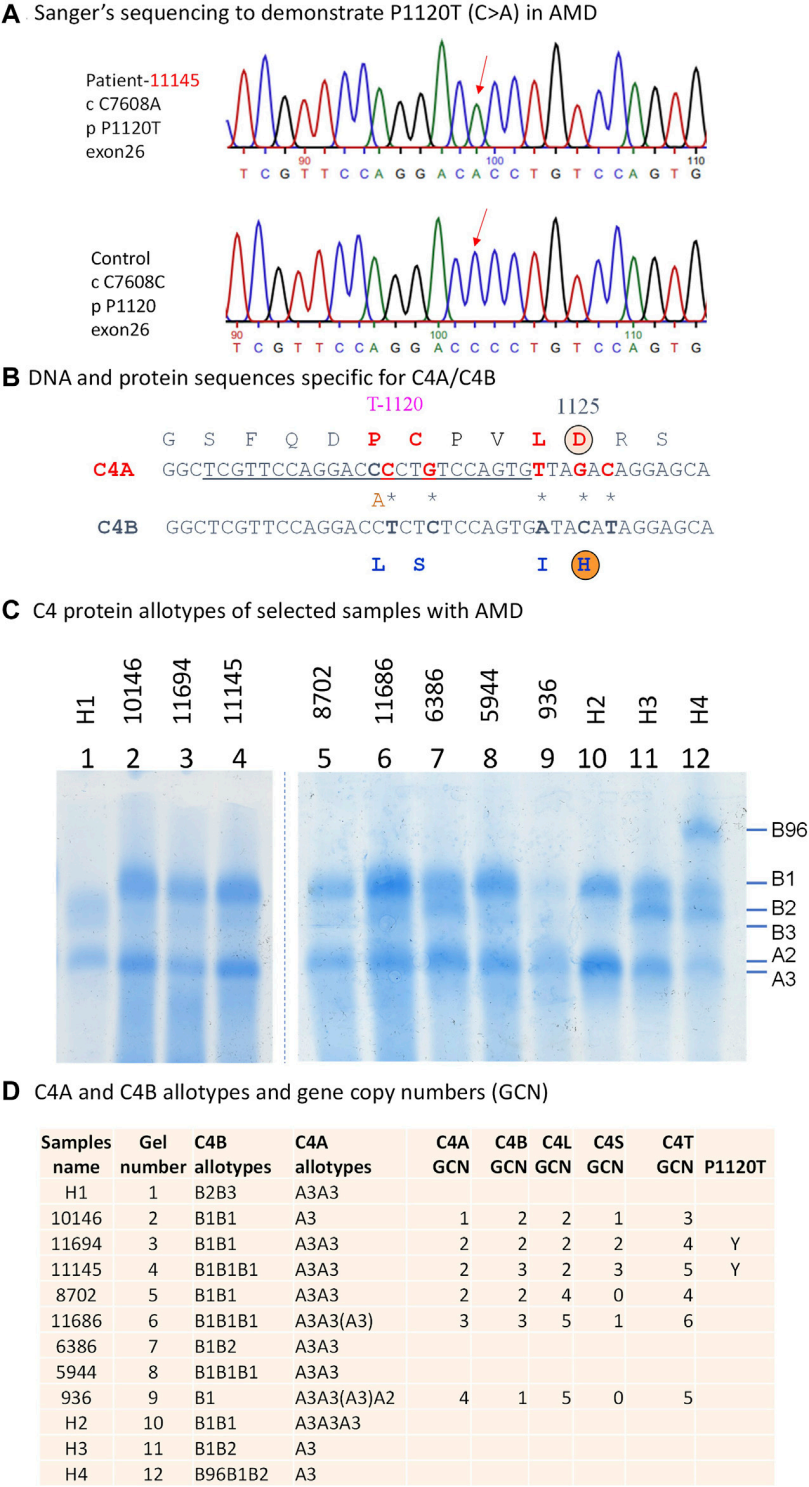
Genotypic and phenotypic analyses of complement C4 in patients with AMD. Genomic sequence analyses of a 2.5 kb region corresponding to exons 22 and 30 encoding the polymorphic C4d region. **(A)** DNA sequences specific for P1120T (DNA: C7608A) variation in a patient with AMD and healthy controls are indicated by red downward arrows. **(B)** A comparison of DNA and amino acid sequences between C4A and C4B at the isotypic region. The charged isotopic residues are circled (D = Aspartic Acid and H = Histidine). The isotypic amino acid sequences for acidic C4A (in red fonts) and basic C4B (in blue fonts) with the P1120T (DNA: C7608A) indicated. **(C)** Immunofixation experiment to show protein polymorphisms of C4A and C4B using EDTA-plasma. H1 to H4 are controls. **(D)** Gene copy number variations and protein phenotypes of complement C4 in eight patients with AMD. Y = Yes for the presence of P1120T.

We identified and cloned the *C4A* genes for two subjects in whom targeted sequencing confirmed the presence of the P1120T variant. Sanger sequencing of individual C4d clones from both subjects revealed a C>A substitution that contributed to the P1120T variation (Figures 3A, B). Immunofixation experiments of EDTA-plasma from those two subjects revealed that this polymorphism did not change the electrophoretic mobility of C4A protein in a standard allotyping gel by immunofixation (Figures 3C, D).

### 3.6 Localization of C4A protein in the human retina

**FIGURE 4:**
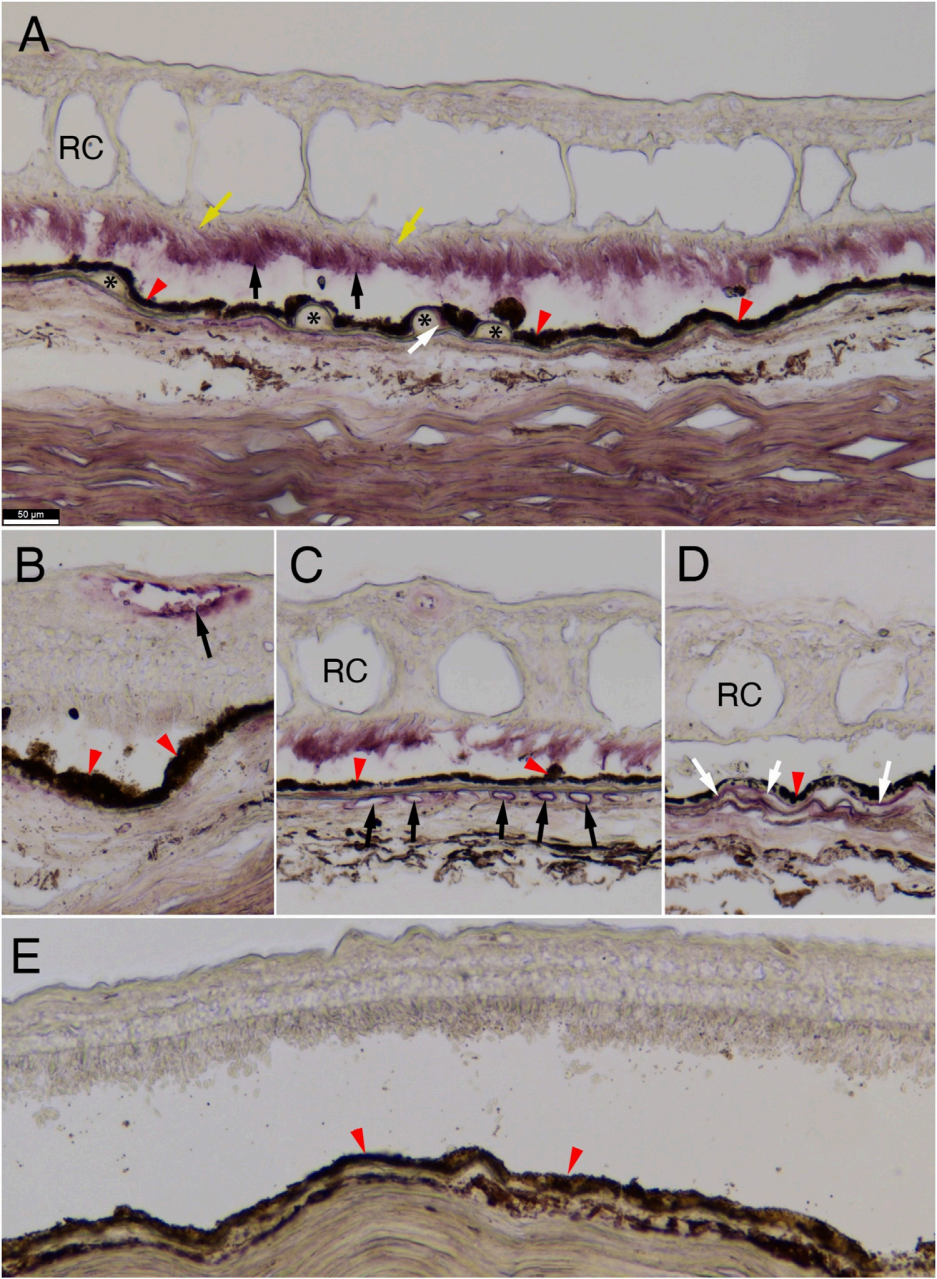
C4A protein immunostaining in human retina. Immunohistochemistry for C4A protein expression on human retinal cross-sections. **(A–D)** Immunohistochemistry on sections of an eye from a 92-year-old-female with neovascular pathology showing accumulation of C4A in photoreceptor outer segments (A: black arrows), at the edge of drusen (A: white arrow), endothelial cells of the retinal and choroidal vasculature (B and C, respectively: black arrows), and in the Bruch’s membrane (D: white arrows; **(A)**: asterisks mark drusen and yellow arrows mark photoreceptor inner segments; **(A–E)**: red arrowheads mark retinal-pigmented epithelium, **(A, C, D)**: RC marks retinal cysts). Of note, photoreceptor outer segment staining **(A)** is not seen in areas of the same eye where retinal cysts (RC) are absent **(B)**. No appreciable staining is seen in a retina from a 78-year-old male subject without AMD **(E)**. Scale bar in **(A)** of 50 µm is the same for all panels. The antibody used was a monoclonal for anti-Rg1 that is usually associated with C4A.

To better understand the role that C4A may play in AMD pathogenesis, we performed immunohistochemistry on retinal cross-sections of a human donor eye with advanced neovascular pathology and a control eye with no eye disease. We used a monoclonal RgD1 that is specific against Rg1, indicating that it is mostly against the C4A protein (Figure 4). C4A accumulation was observed on photoreceptor outer segments in neovascular regions of the retina that contained retinal cysts (Figure 4A). In regions without retinal cysts in the same eye, no C4A was observed on photoreceptor outer segments (Figure 4B). C4A was also observed on the edge of some drusen (Figure 4A) and on endothelial cells of the retinal and choroidal blood vessels (Figures 4B, C), and in the Bruch’s membrane (Figure 4D). No appreciable signal was visualized in the cross-section of the tissues from the subject with no AMD (Figure 4E).

## 4 Discussion

We aimed to identify novel rare or low-frequency variants for AMD by combining targeted sequencing data from two cohorts. The main finding of an association with rs28357075 (V1207A) in *C4A* was replicated at *p* = 0.037 in the UKBB WES data. The *C4A* mutation P1120T, suggested to be protective for AMD in this study, is a novel polymorphism and the first variant of isotypic sequence specific for C4A (Zhou et al., 2021).

Single-variant associations of 11 variants (including 3 novel) and gene-based associations of 10 genes with AMD (including 3 novel) were found. Among the previously discovered variants was well-known AMD risk protein-coding rare variants such as *CFH* R1210C (Raychaudhuri et al., 2011). The 3 novel rare variants had not been identified previously for AMD probably due to their low MAF, uncertain imputation in previous studies, and the complex gene copy number variations of *C4A* and *C4B* (Yang et al., 2007). Of the 8 known AMD risk variants, 6 were present in the UKBB data, and 4 of these 6 were replicated at *p* < 0.05. In comparison with published studies, our investigation had two advantages that facilitated the discovery of new variants. First, the sample size was substantially increased by combining datasets (Seddon et al., 2013; Zhan et al., 2013). Second, directly sequencing coding variants added incremental power over imputed genotypes that had been used in previous studies (Fritsche et al., 2013; Fritsche et al., 2016), especially for low-frequency and rare variants. It also suggests that new variants are likely to be discovered in association with AMD. As subjects in both discovery samples were primarily of European ancestry, the increase in power gained from combining these studies outweighs the impact of population structure differences on our results (Persyn et al., 2018).

Much evidence supports the important role of the complement system in the etiology of AMD. A dysregulated complement pathway may stimulate inflammation thereby accelerating AMD by damaging tissues. However, it may also promote tissue repair or remodeling, a process known as para-inflammation (Medzhitov, 2008; Chen and Xu, 2015). Several common and rare genetic variants in complement genes have been reported to be associated with AMD, including *CFH*, *C2*, *C3*, *CFB*, *CFI* and *C9* (Klein et al., 2005; Maller et al., 2006; Maller et al., 2007; Fagerness et al., 2009; Neale et al., 2010; Raychaudhuri et al., 2011; Fritsche et al., 2013; Seddon et al., 2013; Zhan et al., 2013; Yu et al., 2014; Fritsche et al., 2016; Yu et al., 2016). Thus, much of the pathology and genetic results on the complement system’s role in AMD mostly points to the alternative pathway (AP) activation and, particularly, its regulation by FH and FI as being the key players. However, recent data also indicate that the classical pathway (CP) and the lectin pathway (LP) are probably involved (Anderson et al., 2002; Chen and Xu, 2015). In multiple disease conditions, the initial activation of the complement system is initially driven by LP or by CP engagement but then is substantially amplified by AP activation. In these cases, up to 90% of the C3b deposition is driven by the AP.

C4 plays an important role in the activation of the classical and lectin pathways of the complement system. In both pathways, it is activated and cleaved by serine proteases leading to formation of C4b2a (C3 convertase) which in turn cleaves C3 into C3a and C3b. Together with C2 and factor B, *C4* genes are located in the major histocompatibility complex (MHC) class III region on human chromosome 6 (Carroll et al., 1984). C4 genes and proteins are highly polymorphic, varying in gene copy number, serum protein levels and hemolytic activities as well as in the affinities of substrate binding and ability to elicit immune responses (Coss et al., 2023).

C4A and C4B are the two isotypes of C4. They share 99% protein sequence identity. One study reported that *C4A* gene copy number was lower in AMD patients compared to unaffected controls (Grassmann et al., 2016). In our study, both single-variant and gene-based association analyses suggested that rare variants of C4A protein may be associated with AMD with V1207A as a risk factor and P1120T as a protective factor. The top rare variant (p.V1207A, MAF = 0.0081 in 1,000 genomes and MAF = 0.0082 in ExAC) was a risk variant for AMD in our analyses and independent of reported known AMD risk variants in the *CFB/C2* locus or any other variants that were associated with *C4A* copy number (*r* ^*2*^ > 0.7). The 5 nonsynonymous rare variants in C4A protein were all in the α-chain. The α-chain has a cleavage site by C1s or MASP2 into C4a and C4b. C4b is an anchor protein on which progression of the lectin and classical activation pathways occurs (Zhou et al., 2021; Coss et al., 2023). The typical outcomes of complement activation are formation of the membrane attack complex to destroy immune targets, generation of anaphylatoxins to attract inflammatory cells to sites of complement activation and opsonization of immune aggregates for phagocytosis and removal. Through a yet to be defined mechanism, the processed activation product C4d, which harbors multiple polymorphic amino acid residues, modulates recognition of self and nonself. Robust deposition of C4d fragments on tissue grafts such as in the kidney after transplantation are relevant biomarkers of a pending rejection (Feucht et al., 1991; Feucht, 2003). On red cells they indicate robust complement activation with generation of alloantibodies in patients undergoing blood transfusion (Bohmig et al., 2016). Further, they mediate production of autoantibodies in patients with systemic autoimmune diseases, particularly in the setting of a dysfunctional immune system (Yang et al., 2007).

Our observation using a mAb against Rg1 demonstrates the presence of C4A protein in proximity to drusen formation is consistent with a possible engagement of C4A in the removal of immune complexes, apoptotic or necrotic materials. The enrichment of C4 protein in endothelial cells of the retinal vasculature in AMD and its presence in soft drusen suggests that C4A may be involved in multiple aspects of the disease such as drusen formation, debris removal and neovascularization. However, a definitive conclusion on the selective involvement of C4A awaits future immunostaining experiments using a Ch1-specific mAb (Mauff, 1998).

C4 protein was expressed in the retinal and choroidal vasculature but not in the lesion area of geographic atrophy of human donor eyes (Katschke et al., 2018). They also found C4 proteins in photoreceptor outer segments in a subset of donor eyes. Other analyses of human donor retinas showed that complement proteins C3 and C5 accumulated in the aging eye, with evidence of C3 accumulation in the Bruch’s membrane/choroidal interface of advanced cases of AMD (Anderson et al., 2002; Loyet et al., 2012). Likewise, analysis in induced-pluripotent stem cells derived from RPE demonstrate that many complement proteins show increased expression in response to nitrite alteration of extra-cellular matrix, a modification typical of aging Bruch’s membrane (Gong et al., 2020). Collectively, these analyses could point to altered complement activation coinciding with aging. However, mechanisms by which complement genetic variants and their protein products alter disease risk and progression, such as in drusen formation and clearance of debris, require further investigation.

Defining *C4* variants that are protective and others that are risk factors provides an opportunity to test mechanisms whereby complement activation leads to inflammation, immune clearance and humoral immune responses in patients with AMD. Here we discovered an association between *C4A* (V1207A) and increased risk of AMD and confirmed this finding using UKBB WES data. This V1207A variant is part of the Rg1/Ch1 blood group determinants, VDLL 1207-1210 ADLR, which are mostly associated with C4A and C4B, respectively (Yu et al., 1986; Yu et al., 1988; van den Elsen et al., 2002). The anti-Chido and anti-Rodgers *allo*antibodies are generated when a human subject receives a blood transfusion and there are mismatched polymorphic C4 protein variants between the recipient and the donor (Giles et al., 1976; Yu et al., 1986; Giles et al., 1988; Reilly et al., 1991a).

The phenomenon reflects the binding and deposition of activated C4A and C4B proteins on nearby cell surfaces such as erythrocytes. The polymorphic residues in the C4d region are readily differentiable during the refined process of self/nonself recognition among different individuals during allo-immune responses. The C4d region is engaged in self/nonself differentiation which can be further substantiated by the presence of extensive polymorphisms in that region (Belt et al., 1985; Yu et al., 1986; Zhou et al., 2021). The chemical binding reactivities of the C4 thioester carbonyl group to substrates (after activation) are modulated by the isotypic residues PCPVLD 1120-1125 LSPVIH that are 82-87 amino acids residues upstream of Rg1/Ch1 epitopes (Carroll et al., 1990; Reilly et al., 1991b; Dodds et al., 1996).

The potentially protective role of P1120T in our AMD discovery cohort is intriguing, though it requires further independent replications. Many associated questions emerge, e.g., whether it affects the chemical reactivities of the thioester bond and the differential binding affinities to substrates, and whether it plays a role in humoral immune responses. Our allotyping gels using EDTA-plasma revealed that this mutation did not change the protein’s electrophoretic mobility compared with regular C4A3. Likewise, the association of this variant with AMD (OR = 0.26) failed to replicate in UKBB WES data (OR = 0.90). This variant appears in 0.24% of cases and 0.90% of controls in our samples, as opposed to 0.12% of cases and 0.13% of controls in the UKBB. Although this variant may still affect protein function, it is possible that the protective effect is overestimated in our sample. The *C4* variants reported herein, especially those altering the thioester or C4d domain, can be further explored. A possibility is that such changes alter the ability of C4A to attach to “debris” in AMD for clearance. While the complement C4 story described has genetic and protein data supporting its role in the development of AMD, we cannot rule out the possibility that finding C4A protein in retina in this study may reflect an inflammatory response to neovascularization (Arrigo et al., 2023).

In conclusion, we detected a novel, rare nonsynonymous variant in *C4A* which increased risk of AMD (V1207A), which was replicated in the UKBB WES data. We also found a suggestive association between P1120T and reduced risk of AMD. We further showed that C4A protein was present in soft drusen and endothelial cells of the choroidal and retinal vasculature in human donor tissue with neovascular AMD. This suggests that C4A protein may be involved in or “attracted to” areas with drusen and engaged in the pathogenesis of neovascular disease. Thus, C4A could be a novel target for the treatment and prevention of AMD.

## Data Availability

The original contributions presented in the study are included in the article/Supplementary Material. Further inquiries can be directed to the corresponding author.
